# Technology beyond Biology; Isn’t It Time to Update WHO’s Definition of Health?

**DOI:** 10.3390/medicina60091456

**Published:** 2024-09-05

**Authors:** Maja Baretić, Dragan Primorac, David de Bruijn, Velimir Altabas

**Affiliations:** 1 Department of Endocrinology and Diabetes, Internal Clinic, University Hospital Centre Zagreb, 10000 Zagreb, Croatia; 2 School of Medicine, University of Zagreb, 10000 Zagreb, Croatia; velimir.altabas@gmail.com; 3 St. Catherine Specialty Hospital, 10000 Zagreb, Croatia; draganprimorac2@gmail.com; 4 International Centre for Applied Biological Research, 10000 Zagreb, Croatia; 5 School of Medicine, Josip Juraj Strossmayer University of Osijek, 31000 Osijek, Croatia; 6 Faculty of Dental Medicine and Health, Josip Juraj Strossmayer University of Osijek, 31000 Osijek, Croatia; 7 School of Medicine, University of Split, 21000 Split, Croatia; 8 School of Medicine, University of Rijeka, 51000 Rijeka, Croatia; 9 School of Medicine, University of Mostar, 88000 Mostar, Bosnia and Herzegovina; 10 Eberly College of Science, The Pennsylvania State University, State College, PA 16802, USA; 11 The Henry C. Lee College of Criminal Justice and Forensic Sciences, University of New Haven, West Haven, CT 06516, USA; 12 Regiomed Kliniken, 96450 Coburg, Germany; 13 Department of Philosophy, Auburn University, College of Liberal Arts, 7030 Haley Center, Auburn, AL 36849, USA; dmdebruijn@gmail.com; 14 Sisters of Charity University Hospital Centre, 10000 Zagreb, Croatia

**Keywords:** health, technology, extended health hypothesis, extended mind hypothesis, WHO

## Abstract

Technology is increasingly shaping human life, particularly in healthcare, where recent advancements have revolutionized patient care. Despite these advances, the World Health Organization’s (WHO) definition of health remains rooted in traditional notions, raising questions about its adequacy in light of technological progress. This paper explores the conceptual and practical limitations of the current definition and argues for its revision to encompass the role of technology in health. This paper examines the evolving landscape of healthcare technology and its philosophical implications, drawing on theories such as the Extended Health Hypothesis and the Extended Mind Hypothesis. It claims that health extends beyond traditional biological boundaries and includes the influence of technology on well-being. This paper advocates for a re-examination of the WHO definition of health to reflect the integral role of technology in modern healthcare. Recognizing technology as part of health necessitates a broader conceptual framework that acknowledges the interconnectedness of biology, technology, and human well-being. Given technology’s transformative role in healthcare, this paper argues for a revaluation of the WHO’s definition of health to encapsulate the evolving relationship between technology and human well-being. At the end, we propose a new definition recognizing that health is a dynamic state of physical, mental, social, and technological well-being, wherein individuals can achieve optimal quality of life through the harmonious integration of biological, psychological, and technological factors. This state encompasses not only the absence of disease but also the effective utilization of advanced technologies.

Technology is changing the fundamental dimension of human life, playing an ever-greater role in healthcare. Recent advancements in medical technology have facilitated a symbiotic relationship between technology and patient well-being. The most striking examples include deep neural stimulation, in which targeted electrical impulses are delivered to specific brain regions, modulating neural activity and alleviating symptoms of neurological disorders like Parkinson’s Disease. This technique has significantly advanced the understanding of brain electrical activity, enabling targeted brain stimulation by modifying neurotransmitters in specific areas of the brain. Meta-analyses have shown that this therapeutic approach can greatly reduce motor symptoms such as tremors and rigidity, enhancing the quality of life for many patients who do not respond adequately to medication alone [1]. Similarly, cardiac implantable electronic devices, such as pacemakers and implantable cardioverter-defibrillators, are implanted in the heart to continuously monitor its electrical activity and, when necessary, generate additional electrical stimuli. Furthermore, when abnormal rhythms are detected, some types of these devices may deliver electrical impulses or shocks to correct and restore normal heart rhythms, helping to prevent sudden cardiac death [2]. One step further in technology is artificial intelligence (AI) being used in type 1 diabetes. Machine learning algorithms have revolutionized insulin delivery by continuously analyzing glucose monitoring data and predicting insulin requirements in real-time. An insulin pump equipped with AI for type 1 diabetes can autonomously adjust insulin delivery based on real-time glucose data and predictive algorithms. This advanced system helps maintain optimal blood glucose levels by continuously learning from a patient’s glucose patterns and adapting its insulin dosage to improve overall glycaemic control [3]. Furthermore, brain–computer interfaces offer future possibilities facilitating direct communication between the brain and external devices enabling communication for individuals with paralysis, assisting in motor control for prosthetics, monitoring brain activity for diagnosing neurological disorders, and providing targeted therapies [4]. The implementation of Whole Genome Sequencing (WGS) in clinical practice marks a critical advancement in healthcare. WGS allows for a comprehensive analysis of an individual’s entire genetic makeup, facilitating more precise diagnosis, personalized treatment plans, and the identification of genetic predispositions to certain diseases. This technology has the potential to revolutionize fields such as oncology, where it can identify specific genetic mutations driving cancer, and rare genetic disorders, offering insights that were previously unattainable with traditional genetic testing methods [5].

In the post-pandemic era, where physical interactions are restricted, technology has proven its ability to go beyond traditional healthcare boundaries, offering new dimensions of care through virtual environments and robotic assistance. For example, the integration of social robotic systems with virtual reality environments has provided innovative solutions for delivering healthcare when in-person contact is limited [6]. Additionally, advancements in AI and extended reality have become central to modern healthcare systems, showcasing how these technologies are not just supplementary but essential in redefining health and enhancing overall well-being [7]. However, technology has some disadvantages and side effects that should be considered, like digital and information overload. Constant exposure to digital devices and an excess of notifications and information can lead to feelings of becoming overwhelmed, distraction, and mental fatigue. Another side effect may be social over-comparison leading to feelings of inadequacy, loneliness, low self-esteem, excessive screen time, and sedentary behaviour.

Advancements in technology might influence how we define and perceive various aspects of health. Technology encompasses more than just medical technologies; it also extends to broader influences on lifestyle, well-being, and social determinants of health. For example, with the frequent usage of wearable devices and mobile health apps, patients and healthcare providers can track different aspects of health in real-time, including vital signs, activity levels, nutrition, sleep patterns, and more. Technology provides access to vast amounts of information on health, wellness, and self-improvement. This information may empower individuals to make informed decisions about their well-being, leading to healthier lifestyle choices. Traditional clinical settings like hospitals and outpatient clinics may be transformed through telemedicine, remote patient monitoring, and virtual care platforms, expanding access to care and empowering individuals to manage their health continuously and from anywhere, improving accessibility, convenience, and the continuity of care [8]. In addition, social media, messaging apps, and online communities enable individuals to connect with others, share experiences, and receive support, fostering a sense of belonging and social connection that contributes to well-being. For example, technology offers various tools and resources for managing stress, anxiety, depression, and other mental health issues, including meditation apps, online therapy platforms, and mental wellness resources.

Recognizing the connection between technology and health is necessary to understand health beyond traditional clinical viewpoints. As a result, there may be a greater emphasis on preventive and predictive approaches to healthcare, aiming to intervene earlier and mitigate health risks before they escalate. In addition, advances in genomics, precision medicine, and AI enable the customization of healthcare interventions based on an individual’s unique genetic makeup, lifestyle factors, and health history. Such a personalized approach may redefine health as not only the absence of disease but also the optimization of each person’s biological and physiological state.

Nevertheless, in practice, the World Health Organization (WHO) seems to continue to rely on an increasingly outmoded definition of health, which holds that … “health is a state of complete physical, mental and social well-being, and it does not merely describe the absence of disease or infirmity [9].” In many cases, it was stressed that this definition underscores the holistic nature of health, emphasizing not only the absence of illness but also the presence of positive physical, mental, and social factors contributing to overall well-being. Is it enough? The conceptual and practical limitations of the original definition of health, established in 1948, have become apparent over time. In 1986, the Ottawa Charter for Health Promotion expanded this definition by emphasizing health as a resource for daily life rather than merely a goal. It underscored that health is a dynamic resource, enabling individuals to navigate daily challenges and contribute to their communities. By 2000, there was a growing emphasis on equity, the social determinants of health, and the significance of mental and emotional well-being. The evolving definition by the WHO reflects a broader understanding of health, recognizing its multifaceted nature and the need to integrate various aspects of well-being and social factors, though it does not yet fully incorporate the role of technology.

There are other definitions of health and none of them include technology. The American Medical Association (AMA) claims that health is “the state of being free from illness or injury,” focusing on the absence of disease and functional well-being; the National Institutes of Health (NIH) says that health is described as “the level of functional or metabolic efficiency of a living being”, which includes the body’s ability to maintain homeostasis and adapt to stressors; and some functional health models observe health as the “ability to perform daily activities and roles effectively,” focusing on functional capacity and quality of life and social determinants according to which health is defined in terms of the “conditions in which people are born, grow, live, work, and age”, highlighting the impact of social, economic, and environmental factors [10].

Bearing in mind technological advances in the years that have passed since the WHO definition, it is time for medical practice to consider certain fundamental changes in theoretical thinking concerning the concept of health.

In recent decades, critics have argued that the WHO’s definition of health does not adequately reflect the significant impact of digital technologies on well-being. Sherry Turkle (2011) argues that the WHO definition overlooks the psychological and social impacts of technology, such as its effects on mental well-being and social connections [11]. Luciano Floridi (2014) critiques the definition for not reflecting how digital technologies reshape our understanding of health and reality [12]. Nick Srnicek (2016) highlights that the definition fails to address how digital platforms influence economic and social aspects of health [13]. Julie B. Cohen (2019) critiques the definition for not considering the effects of digital environments on privacy and autonomy [14]. Similarly, S. Scott and J. C. S. Sweeney (2020) argue that the WHO definition overlooks the significant role of digital health technologies, such as apps and telemedicine, in managing and enhancing health. We advocate for a revised definition that incorporates these technological dimensions [15].

Possible counterarguments to incorporating technology into the WHO definition of health include concerns that this expansion might overemphasize technological solutions at the expense of fundamental human factors. While technology brings significant advancements, it can also worsen health disparities and introduce new forms of inequality, such as digital divides and privacy concerns, and narrow the focus of healthcare, shifting attention away from critical elements like social support systems and environmental factors that are vital for overall well-being. Furthermore, some may worry that integrating technology into the definition of health could lead to an overreliance on technological solutions, potentially sidelining non-technological approaches to health improvement. This could lead to prioritizing high-tech interventions over more accessible, community-based strategies essential for inclusive healthcare. The answer to sceptics is that technology in healthcare enhances health across the three dimensions of the traditional definition: physical, mental, and social well-being. It aims to improve those aspects.

Although WHO maintains its definition of health, the 11th Revision of the International Classification of Diseases (ICD-11) acknowledges the evolving role of technology in healthcare. ICD-11 includes a chapter featuring Extension codes that provide additional details on diseases. Among these, some codes focus on issues arising from the interaction between humans and medical devices. For instance, *XE5DG* addresses human–device interface problems, *XE6GS* describes patient–device incompatibility, and *XE7ZE* is a part of biocompatibility issues [16].

The concept of health benefits from philosophical and ethical exploration. Traditional views in bioethics and the philosophy of medicine have aligned around the idea of a fixed, individual organism as the focus of health and disease. The “Extended Health Hypothesis” suggests that health and disease are influenced not only by internal factors [17]. In this theory, the concept of health also extends beyond the traditional biological boundaries of the individual. Likewise, it claims that the subject’s health can include elements beyond the organic material, as these are functionally critical to patients’ vital functions. Technology is not merely significant to a patient’s health but has become a part of their health. 

The Extended Health Hypothesis follows from the much-discussed philosophy, the “Extended Mind Hypothesis”, which holds that elements outside the brain or body can be critical parts of cognitive functioning [18]. This hypothesis challenges traditional ideas of cognition and intelligence by suggesting that the human mind is not confined to the boundaries of the skull but extends into the environment through tools, like technology. The introductory question in this hypothesis is Where does the mind stop, and the rest of the world begin? The answer is not only skin and skull but technology too. The previously mentioned brain–computer interface initiates actions based on a person’s thoughts (intentions), translating mental impulses into tangible movements [19]. With the help of technology, the power of thought is transformed into the ability to move objects, which aligns with the philosophical hypothesis of the extended mind. The “Extended Health Hypothesis” and the “Extended Mind Hypothesis” raise questions about whether technology forms an integral part of patient health, and whether device malfunctions should be regarded as a manifestation of disease. We claim that there is health beyond biology, well-being beyond the skin, and thought beyond the skull. In addition, a whole new dimension of health may now be understood as the optimal use of technological innovations to enhance not only the prevention, diagnosis, and targeted treatment of diseases but also to promote overall quality of life.

Traditional conceptions of health, rooted in historical and biological perspectives, have given rise to various medical and philosophical questions. One such issue is how genetic interventions challenge conventional concepts of health, prompting debates on fairness, responsibility, and the distinction between therapy and enhancement [20]. Similarly, the ethical and social implications of enhancing human traits through technology raise concerns about the risks associated with the improvement of human beings [21]. In the realm of AI, the ethical challenges in healthcare emphasize the need to integrate AI’s capabilities with a human-centred approach, ensuring that technology supports rather than undermines the relational aspects of health and healing [22]. Additionally, the transhumanist movement, which advocates for the use of technology to enhance human capacities and transcend biological limitations, explores the philosophical and ethical implications of extending human life, intelligence, and well-being through technological advancements [23].

This paper’s interpretation of the Extended Health Hypothesis resonates with broader discussions in bioethics and enhancement technologies. For instance, John Harris advocates for the ethical use of technology to enhance human abilities and views it as a moral obligation [24]. While this paper aligns with Harris on the potential of technology to improve life, it advances the argument by integrating technology directly into the very definition of health, thus broadening the concept. In contrast, Carl Elliott critiques the medicalization of normal traits through technology, warning against reducing complex human experiences to simple technical issues [25]. This paper departs from Elliott’s cautiousness by embracing technology as a core component of health, arguing that it enriches rather than diminishes the human experience. Similarly, Mary Midgley raises concerns about the ethical implications of biotechnological advancement [26]. While this paper shares her ethical concerns, it diverges by advocating for a redefinition of health that fully incorporates technological advancements as essential to our understanding of well-being.

Integrating technology into the concept of health is redefining and expanding our understanding of well-being. One example is the development of human digital twins, the reproduction of real-world human beings in cyberspace, mirroring their physical and biological characteristics, behaviours, and even certain aspects of their environment. This digital counterpart is created using advanced technologies such as AI, machine learning, big data analytics, and the Internet of Things. By collecting and analyzing data from various sources, including medical records, wearable devices, genetic information, and lifestyle choices, a human digital twin can simulate how an individual’s body might react to different scenarios, treatments, or lifestyle changes. The concept of a human digital twin has significant potential in healthcare. For example, it could allow doctors to personalize treatment plans by testing various interventions on the digital twin before applying them to the patient, thereby reducing risks and improving outcomes. Additionally, human digital twins could help in the early detection of diseases by monitoring and predicting health changes in real-time, opening new perspectives in preventive precision medicine [27]. For example, in patients with conditions like type 1 diabetes, these digital twins go beyond glucose levels, incorporating physiological (i.e., heart rate, blood pressure, motion sensor, hormone level), environmental (i.e., location, air pollution), and even personality factors, allowing better short-term and long-term outcomes [28].

In the future, virtual space and AI will include more than biology and technology. Today, we are witnessing that rapidly evolving personalized medicine approach, which uses multi-OMICS molecular information, exposome, and phenome data, become a reality.

A technology–human unit is a new form of symbiosis. Consequently, revisiting the WHO definition of health to incorporate technology becomes imperative with the word technology in it [29]. Technology has the potential to reshape our understanding of health by providing new tools. It is essential to consider the ethical, social, and equity implications of these technological advancements to ensure that they contribute positively to the health and well-being of individuals and communities.

This expanded definition acknowledges that technology, including digital health solutions, AI, telemedicine, wearable devices, and multi-OMICS, particularly genomic medicine, among others, empowers individuals to engage in their health management actively, helps the early detection of health issues, enables personalized treatment plans, and fosters equitable access to healthcare services globally. Following a patient’s genetic profile, prediction can precede prevention. Technology’s impact on well-being depends on various factors, including individual usage patterns, attitudes towards technology, and the broader socio-cultural context. By promoting a mindful and balanced use of technology, fostering digital literacy, and prioritizing human connection and well-being, we can harness technology’s benefits while mitigating its potential negative effects on overall well-being. Furthermore, and of the highest importance, health encompasses the ethical and responsible development and deployment of technology, ensuring that innovation aligns with principles of equity, inclusivity, privacy, and sustainability. By integrating technological advances into our understanding of health, we recognize the dynamic relationship between human well-being and the evolving landscape of science and technology. 

In summary, this manuscript highlights the evolution of the concept of health, moving from a static definition centred on the absence of disease to a more dynamic and inclusive understanding. Over time, health has come to encompass mental, emotional, and social well-being, with modern considerations now including the role of technology such as AI and medical devices in enhancing health. Incorporating technology into health definitions raises important ethical, social, and political concerns, such as privacy, equity, and the need for new regulatory frameworks.

To adapt to contemporary realities, future research should explore how emerging technologies can be better integrated into our understanding of health. Policymakers must ensure these advancements are accessible and equitable, while interdisciplinary collaboration is essential for developing a comprehensive framework. We propose a new definition: Health is a dynamic state of physical, mental, social, and technological well-being, where individuals achieve optimal quality of life through the integration of biological, psychological, and technological factors. This definition embraces not just the absence of disease but also the effective use of advanced technologies.

## Data Availability

No new data were analyzed during this study.
